# Understanding vulnerabilities and resilience in binge eating disorder among female population and transgender individuals across the lifespan: a narrative review

**DOI:** 10.1007/s40519-026-01891-z

**Published:** 2026-07-24

**Authors:** Nagaia Madini, Chiara Breda, Dana El Masri, Cecilia Ricciardi Rizzo, Simone Lanati, Fatima Cody Stanford, Alessandra Vincenti, Hellas Cena, Rachele De Giuseppe

**Affiliations:** 1 Laboratory of Dietetics and Clinical Nutrition, Department of Public Health, Experimental and Forensic Medicine, University of Pavia, 27100 Pavia, Italy; 2 Clinical Nutrition Unit, Istituti Clinici Scientifici Maugeri IRCCS, Pavia, Italy; 3 MGH Weight Center, Department of Medicine-Division of Endocrinology-Neuroendocrine, Department of Pediatrics-Division of Endocrinology, Boston Area Nutrition Obesity Research Center, Massachusetts General Hospital, Harvard Medical School, Boston, MA USA

**Keywords:** Women, Binge eating disorder, Gender transition, Children, Adolescents, Pregnancy, Menopausal and postmenopausal women

## Abstract

Binge eating disorder (BED) represents a significant public health concern due to its multifaceted physical, psychological, and economic consequences, all of which substantially impair the quality of life of affected individuals. Individuals with BED often experience persistent body dissatisfaction, emotional distress, anxiety, depression, chronic stress, and social prejudice. Fear of judgment, stigmatisation, and discrimination frequently leads to delays in, or complete avoidance of, seeking medical or psychological support. Although BED remains largely underdiagnosed, existing literature indicates a higher prevalence among females. Nevertheless, the factors contributing to its onset remain insufficiently explored, despite the well-documented vulnerability of women throughout various life stages. Specifically, gendered social roles and ongoing hormonal fluctuations are known to influence emotional regulation, potentially triggering and sustaining binge eating behaviours. This narrative review explores the prevalence and associated factors of BED in the female population across the lifespan, including childhood, adolescence, pregnancy, and menopause. It also considers the transgender population, acknowledging how gender identity, stigma, discrimination, and hormonal treatments can influence BED risk and presentation. Moreover, studies highlighted that the risk and maintenance of BED are exacerbated at various life stages by biological changes associated with sex, as well as by sociocultural influences related to gender roles and socially constructed ideals of femininity. Understanding these diverse factors is essential for developing targeted prevention and treatment strategies that address the specific psychological and biological, as well as social challenges faced by women and individuals assigned female at birth. Importantly, further research is warranted to reduce disparities in healthcare access and to dismantle structural barriers and stigma. Both clinical practice and research must embrace inclusive frameworks that acknowledge the unique vulnerabilities of marginalised populations, while also enhancing healthcare professionals’ training to ensure equitable, culturally competent, and effective care.

## Background

Binge eating (BE) represents a maladaptive eating behaviour characterised by the consumption of an unusually large amount of food in a discrete period, accompanied by a perceived loss of control overeating [1, 2]. While BE itself does not necessarily fulfil the criteria for a clinical diagnosis, it may constitute an important risk factor or prodromal phase of a more severe condition.

Binge eating disorder (BED) is characterised by recurrent episodes of eating large quantities of food, often rapidly and to the point of discomfort, accompanied by feelings of loss of control and significant distress, according to the Diagnostic and Statistical Manual of Mental Disorders (DSM-5) of the American Psychiatric Association (APA) [2].

BED constitutes a significant public health concern due to its profound psychological, physical, and economic consequences [3]. Psychologically, BED is closely associated with conditions such as depression, anxiety, and low self-esteem [3–5]. These issues not only reduce an individual's quality of life but also lead to persistent emotional distress and impaired well-being [6]. Beyond individual psychological factors, genetic and sociocultural influences play a crucial role in the development of BED [7–9]. Many individuals with BED struggle with body dissatisfaction, which is often worsened by societal beauty standards and the internalisation of the thin ideal. Exposure to unrealistic body portrayals in media encourages restrictive dieting, which often results in binge eating episodes to compensate for deprivation [10–12]. Additionally, emotional distress acts as both a trigger and a maintenance factor for BED. Feelings of anxiety, depression, and chronic stress contribute to binge eating behaviours, as individuals use food as a maladaptive coping mechanism [13]. Weight stigma and discrimination further reinforce these behaviours, increasing feelings of guilt, shame, and social withdrawal [14]. People with BED frequently face judgments based on body size, which worsen low self-esteem and create barriers to seeking professional help [15, 16].

BED affects both men and women, but there are notable differences in prevalence between the sexes [17]. It is also important to underline that in clinical research, “sex” is traditionally defined as a construct determined by individual genetic features and physiological and anatomical sex traits, while “gender” refers to social norms and roles, which are defined by what society believes is adequate for people based on their sex assigned at birth [18]. Gender is increasingly recognised not as a binary construct, but as a spectrum and can also be described as “someone’s personal and deeply felt internal sense of the self” [18–20].

Women are particularly vulnerable to BED due to factors that include hormonal fluctuations associated with menstrual cycles, pregnancy, and menopause [21, 22]. These hormonal changes can affect emotional regulation and eating behaviours, creating conditions that may facilitate the onset of BED [23]. Additionally, societal pressures related to gender roles amplify these biological susceptibilities [24, 25]. The societal emphasis on unrealistic beauty standards and the idealisation of thinness disproportionately affects women, fostering body dissatisfaction and increasing the likelihood of disordered eating behaviours [26]. Despite being less prevalent in men, BED is often underreported in the male population, leading to a misperception of the true prevalence of BED among men [27, 28]. Men may be less likely to seek help due to stigma, societal expectations of masculinity, or a lack of awareness that their eating behaviours constitute a disorder [29, 30].

Moreover, given the strong interplay between gender identity and mental health, the present review adopts an integrated sex- and gender-based approach. While several BED risk factors are linked to female biological and reproductive transitions, gender-related experiences such as stigma, body dissatisfaction, discrimination, and minority stress may also significantly influence eating behaviours and psychological vulnerability. Therefore, considering both cisgender and transgender individuals allows for a broader understanding of how biological and gender-related factors interact across the lifespan in shaping BE risk [19]. Finally, despite the growing body of literature on BED and disordered eating behaviours in women, existing reviews have primarily focused on specific life stages or selected populations, such as adolescents, pregnant women, or menopausal individuals, without providing an integrated lifespan perspective.

Therefore, this narrative review aims to identify the prevalence of BED and associated factors both in the female population during their lifespan and in transgender population.

## Materials and methods

This narrative review was conducted following predefined inclusion and exclusion criteria to ensure consistency and transparency in study selection. Studies were included if they assessed or screened BED according to DSM-IV (including EDNOS) or DSM-5 diagnostic criteria, or used validated screening instruments for BE. Eligible studies were required to report data on BED prevalence and/or associated risk and protective factors. Only human studies were considered. The review focused on female populations and, where applicable, on gender transition populations (female-to-male). Age-specific populations were included depending on the thematic section of the review, namely children and adolescents (6–24 years), pregnant women, and peri-/post-menopausal women. Only articles published in English within the last 20 years were considered. All study designs were eligible except narrative reviews. These criteria were applied consistently across all sections of the manuscript to ensure a structured and homogeneous synthesis of the available evidence. A total of 28 studies were included in this review.

## Results

### Binge eating disorder in female school-aged children and adolescents

BED is prevalent in children and adolescents and is associated with negative health outcomes and both physical and psychological impairments [31, 32]. Therefore, these life stages represent an important developmental period in which understanding the risk and protective factors for BED can enable the implementation of preventive strategies [31]. The present paragraph describes findings from seven research articles investigating risk and protective factors for binge eating in children, adolescents, and young adults aged 6–24 (Table 1).

**Table 1:** Female school-aged children and adolescents

Author and year	Type of study	Aim	Population	BED diagnosis/screening	Results	Prevalence	Associated factors
Cella S et al., 2021 [31]	Cross-sectional	To test, separate path models for girls and boys examining whether dimensions of body (dis)investment (feelings towards the body, physical touch, body care, and body protection) mediate the relationship between self-esteem and BE, and whether age moderates these associations	1046 (574 M/472F); age range: 11–19 years	BES	▪ Body disinvestment mediates the relationship between self-esteem and BE in both sexes ▪ Absence of body protection and negative attitudes towards it explain more than 2% of the variance in BE, regardless of gender	*n.r*	▪ Low self-esteem ▪ Absence of bodily self-protection ▪ Negative feelings and attitudes towards the body ▪ High BMI
Dakanalis AS et al., 2015 [35]	Longitudinal	To explore the developmental effects of internalising media ideals and self-objectification on negative feelings towards the body and eating disorders in adolescents	685 (324 M/361F); age range: 14–15 years	EDE (Italian version)	The internalisation of media ideals promotes self-objectification, which in turn fuels negative emotions about one’s body and appearance. These emotions may trigger restrictive eating behaviours and binge-eating episodes, leading to a mutually reinforcing cycle of eating disorder symptoms	*n.r*	▪ Media-ideal internalisation ▪ Self-objectification ▪ Body shame ▪ Appearance anxiety ▪ Dietary restraint ▪ Higher BMI ▪ Depressive symptoms ▪ Body image concerns
Pace CS et al., 2021 [39]	Cross-sectional	To compare adolescents with and without BE concerning attachment and alexithymia, and to explore the independent role of these factors in predisposition to binge eating	44 F; age range: 14–18 years	BES	The BE group showed higher BES scores and more insecure–preoccupied attachment than the non-BE group, with no differences in alexithymia. Higher BES scores were associated with lower secure attachment and higher rejecting and preoccupied attachment, as well as higher DIF and TAS-20 scores	9.4%	▪ Insecure attachment representations ▪ Preoccupied attachment pattern ▪ Dismissing attachment pattern
Pace CS et al., 2022 [40]	Cross-sectional	To compare community girls at risk and not at risk for BE in attachment representations through a narrative interview and to test the predictive role of attachment pattern(s) on the risk of BE among community girls	112F; age range 14–18 years	BES	BEG scored higher than NBEG in insecure-preoccupied patterns and anger towards mother, and lower scores in relational coherence, reflective functioning regarding feelings towards father, mother as secure base, self-esteem, and adaptive responses. In the second phase of the study, higher BES scores were found to be correlated with higher scores in the rejecting (r = 0.21, p = 0.014), worried (r = 0.34, p < 0.001) and disorganised (r = 0.18, p = 0.03) patterns. Coefficient analysis revealed that the refusing and worried patterns are significant independent predictors of higher BES scores, with the worried pattern as the strongest predictor	10.85%	▪ Insecure-preoccupied and insecure-dismissing attachment ▪ Lower narrative coherence ▪ Weaker maternal secure base ▪ Reduced reflective functioning and adaptive responses ▪ Greater anger towards mother
Stice E et al., 2017 [32]	Longitudinal	To identify risk factors that predict the future onset of specific EDs subtypes, as well as core symptom dimensions that transcend diagnostic categories	1272F; mean age: 18.5 years, standard deviation: ± 4.2 years	DSM-5	▪ The high-risk sample showed elevated scores in body dissatisfaction (+ 0.6 SD), thin-ideal internalisation (+ 0.6 SD), dieting (+ 0.7 SD), and overeating (+ 1.2 SD) compared to a representative sample ▪ Diagnostic progression was highest for BED (46%) ▪ Multiple baseline risk factors (e.g. body dissatisfaction, negative affect, dieting, overeating) predicted the onset of EDs and symptoms ▪ Multivariate models confirmed unique predictors for each disorder (e.g. body dissatisfaction and overeating for BED) ▪ Predictors for core symptoms (e.g. binge eating, compensatory behaviours, overvaluation of weight/shape) included dieting, body dissatisfaction, negative affect, and overeating	▪ BED: 5.5% ▪ Subthreshold: BE 5.6% ▪ Recurrent BE symptoms: 29.0%	▪ Functional impairment ▪ Thin-ideal internalisation ▪ Body dissatisfaction ▪ Overeating
Yamamiya Y et al., 2023 [34]	Longitudinal	▪ To characterise the sequence of prodromal symptom emergence in adolescents who developed threshold/subthreshold Eds (e.g. AN, BN, BED, or PD) during a 7-year follow-up (primary aim) ▪ To test whether prodromal symptoms increased the risk of subsequent onset of each eating disorder type, focusing on which prodromal symptoms had the strongest predictive effects for each disorder (secondary aim)	496F; mean age 13.02 years, standard deviation: ± 0.73 years	DSM-5	**Incidence over 7 years,** BED: 13 cases (12 subthreshold, 3 threshold) **Symptom emergence patterns,** ▪ Compensatory behaviours typically emerged before binge eating (e.g. 53.8% for BED) ▪ Weight/shape overvaluation was the most common first cognitive symptom (32.0%–62.5%) ▪ Cognitive symptoms generally preceded behavioural ones (84.6% for BED) **Significant univariate predictors,** BED: Overvaluation only (OR = 6.40; SRD = 0.38) **Predictive accuracy of prodromal symptoms,** 87% for BED	*n.r*	▪ Weight/shape overvaluation
Herle M et al., 2020 [39]	Longitudinal	To investigate the associations between childhood eating behaviours during the first ten years of life and EDs behaviours and diagnoses at 16 years	4,760 (1956 M/2804F); 1.3 years old	BE: Questionnaire items from the Youth Risk Behaviour Surveillance System; BED: DSM-5	Overeating was associated with increased risk of adolescent BE (RD: 7%; 95% CI: 2, 12) and BED (RD: 1%; 95%CI: 0.2, 3)	BE: 15.8%; BED: 1.5%	▪ Childhood late-increasing overeating trajectory ▪ Childhood early-increasing overeating trajectory

For brevity and consistency with the aim of this narrative review, only findings related to BED and BE in the female population are reported

BED, binge eating disorder; BE, binge eating; EDs, eating disorders, EDE, Eating Disorder Examination; TAS-20, Toronto Alexithymia Scale; DIF, difficulties in identifying feelings; BEG, binge eating group; NBEG, non-binge eating group; BMI, body mass index; AN, anorexia nervosa; dlPFC, dorsolateral prefrontal; DSM-5, Diagnostic and Statistical Manual of Mental Disorders-Fifth Edition; PD, purging disorder; n.r., not reported

In a study conducted by Cella and colleagues [33], 1,046 Italian students were examined to assess whether feelings and attitudes towards the body (specifically comfort with physical touch, body care, and body protection) mediated the relationship between self-esteem and BE behaviour. Paying particular attention to the female population, the study found a significant direct effect of self-esteem on BE, which was partially mediated by body image feelings and body protection [33]. It follows that body investment (a less protective attitude towards one’s own body and a sense of inadequacy fuelled by an intensification of pubertal impulses, alongside body dissatisfaction), is a critical factor in the development of BE in female adolescents and should be integrated into etiological models of the behaviour [33].

Other risk factors collectively contribute to the development and maintenance of BED in adolescents include (i) **the pursuit of the thin beauty ideal** leading to body dissatisfaction, which can result in unhealthy weight control behaviours and trigger BE as a response to deprivation or emotional distress; (ii) **the negative affect**, including feelings of sadness, anxiety, and low mood, increasing vulnerability to BE by driving individuals to use food as a coping mechanism; and (iii) **the impaired interpersonal functioning** (e.g. social isolation or strained relationships), exacerbating the risk by intensifying feelings of loneliness and emotional distress. Thus, Stice and colleagues [34] combined longitudinal data from one efficacy trial and two effectiveness trials, to examine risk factors that predict the future onset of EDs, as well as core symptom dimensions that crosscut disorders, on 1272 female adolescents. Regarding BED, results showed that among participants, 5.5% presented BED; 5.6% presented subthreshold BE; and 29% reported recurrent BE symptoms [34]. Moreover, significant risk factors for subthreshold BED included elevated thin-ideal internalisation, body dissatisfaction, and functional impairment, whereas BED was associated with body dissatisfaction, overeating, and functional impairment [34] (see Tables 2, 3, 4).

**Table 2:** Pregnancy

Author and year	Type of study	Aim	Population	BED diagnosis/screening	Results	Prevalence	Associated factors
Bye A. et al., 2020 [42]	cross-sectional	To estimate prevalence of lifetime and current eating disorders (EDs) in a sample of pregnant women in South-East London and to describe their socio-demographic and clinical characteristics	n = 545 pregnant women between November 2014–June 2016	Researchers administered the structured clinical interview for DSM-IV and DSM-IV-TR Axis I (SCID-I-Research Version; First, Spitzer, Gibbon, & Williams, 2002) and Axis II disorders (SCID-II; First, Gibbon, Spitzer, Williams, & Benjamin, 1997)	Weighted prevalence of lifetime EDs was 15.35% (95% [CI] [11.80, 19.71]), and current EDs was 1.47% (95% CI [0.64, 3.35]) Depression, anxiety, and history of deliberate self-harm or attempted suicide were common in pregnant women with EDs. Identification of EDs in antenatal care was low	▪ Lifetime prevalence of BED 1.67% ▪ Current BED prevalence 0.51%	▪ White ethnicity ▪ Higher education ▪ Non-cohabiting relationship ▪ Pre-pregnancy underweight/ overweight BMI ▪ Current depression, anxiety, borderline personality disorder ▪ History of deliberate self-harm/suicide attempts
Çiçekoğlu Öztürk P et al., 2024 [41]	Systematic review and meta-analysis	To reveal the prevalence of eating disorders (EDs) and related factors in pregnancy	11 studies involving 2,369,520 pregnant women	The diagnosis of EDs was selected according to the most recent DSM-5 classification	The prevalence of BED during pregnancy is higher than that of pre-pregnancy. The prevalence of EDs in general is higher in pregnant women under the age of 30, have higher educational levels, married, and with a normal BMI BED is the most common eating disorder in pregnant women	▪ BED prevalence during pregnancy: 3.8% ▪ BED prevalence before pregnancy: 3.3%	▪ Younger maternal age (< 30) ▪ Secondary education ▪ Married status ▪ Normal BMI ▪ Comorbid anxiety and depression
Knoph Berg C et al., 2011 [46]	Prospective-cohort study	To identify factors associated with incidence and course of broadly defined BED in pregnancy	n = 45,644 pregnant women	Questionnaire included several items on EDs symptoms and behaviours that were designed in accordance with the criteria for eating disorders in the DSM-IV	Incidence of BED was significantly associated with lifetime sexual abuse, lifetime physical abuse, lifetime major depression, symptoms of anxiety and depression, low life satisfaction, low self-esteem, low partner relationship satisfaction, smoking, alcohol use, lack of social support, and several weight-related factors. Continuation was negatively associated with thoughts of being overweight before pregnancy. Remission was positively associated with thoughts of being overweight before pregnancy and negatively associated with overvaluation of weight	The prevalence of broadly defined BED in early pregnancy was about 4.8% of women	▪ Sexual/physical abuse ▪ Depression/anxiety ▪ Low life satisfaction, self-esteem, partner support ▪ Smoking, alcohol ▪ Low social support ▪ weight concerns
Silvani J et al., 2020 [45]	Multicenter cohort study	To evaluate the relationship of BE in pregnancy with gestational weight gain, BE at postpartum and postpartum weight retention in women with GDM	n = 1958 pregnant women with GDM	DSM-5	Prevalence of BE was 31.6% (95% CI 29.5–33.6%) during pregnancy and 30.0% (95% CI 28.0–32.1%) at postpartum. The risk of exceeding the IOM's recommendation for gestational weight gain was 45% higher (RR 1.45, 95% CI 1.29–1.63) in women who had BE during pregnancy compared to those who did not. The risk of having postpartum weight retention above the 75th percentile was 33% higher (RR 1.33, 95% CI 1.10–1.59) among those with BE compared to those without	BE prevalence: 31.6%	▪ Excessive gestational weight gain (above IOM recommendations) ▪ High postpartum weight retention (above 75th percentile)

For brevity and consistency with the aim of this narrative review, only findings related to BED and BE in the female population are reported

DSM-IV, Diagnostic and Statistical Manual of Mental Disorders-IV; DSM-IV-TR, Diagnostic and Statistical Manual of Mental Disorders-IV—Revised Text; DSM-5, Diagnostic and Statistical Manual of Mental Disorders-Fifth Edition; EDs, eating disorders; BE, binge eating; BED, binge eating disorder; GWG, gestational weight gain; AN, anorexia nervosa; BN, bulimia nervosa; CI, confidence interval; GDM, gestational diabetes mellitus; RR, risk ratio; OR, odds ratio; SCID, Structured Clinical Interview

**Table 3:** Menopausal and post-menopausal women

Author and year	Type of study	Aim	Population	BED diagnosis/screening	Results	Prevalence	Associated factors
Mangweth-Matzek B et al., 2014 [49]	Cross-sectional study	Assessing prevalence and correlates of EDs in middle-aged women	715 community women, aged 40–60 years	German version of the SCID	The EDs group had lower educational level and more frequent non-European origin than the NE (normal eating) group Women reporting EDs showed significantly higher scores on the EDI total score and subscales (Drive for Thinness; Bulimia; Body Dissatisfaction) They demonstrated higher scores on the CES-D and on the BSQ)	BED prevalence = 1.54% Subthreshold BE with loss of control prevalence = 2.66%	▪ Subthreshold eating disorder status ▪ Loss-of-control BE ▪ Comparable psychopathology to full ED ▪ Impaired quality of life ▪ Body image disturbance
Wilfred SA et al., 2021 [63] **Sample 1**	Cross-sectional study	Assessing BE prevalence, frequency, and health correlates among older women	185 women ranged from 60 to 83 years of age	VA-BES	BE frequency was correlated with higher BMI, more EDs symptoms, greater negative affect, and less frequent consumption of nutritious foods BE resulted in a risk factor for obese status based on self-reported BMI (OR = 2.08; 95% CI [1.52; 2.82], p < 0.001)	BE (at least weekly) prevalence = 26.5%	▪ Higher negative mood ▪ Greater worry ▪ Higher BMI ▪ Less nutritious food consumption
Wilfred SA et al., 2021 [63] Sample 2	Cross-sectional study	Assessing BE prevalence, frequency, and health correlates among older women	64 older women, aged 66 years old and above	EDDS-5	BE frequency was correlated with higher anxiety and internalised weight stigma	BE (at least weekly) prevalence = 20.4%	
Wilfred SA et al., 2021 [63] Sample 3	Cross-sectional study	Assessing BE prevalence, frequency, and health correlates among older women	100 women aged from 55 to 79 years	EDE-Q	BE frequency was positively associated with greater depressive symptoms and higher body shame. BE frequency resulted in a risk factor for obesity (OR = 1.12; 95% CI [1.00, 1.24], p = 0.04)	BE (at least weekly) prevalence = 19%	
Kilpela LS et al., 2023 [64]	Cross-sectional study	Examining the characteristics of older women presenting with BE in later-life, including age of onset, frequency of BE, and qualitative distress	71 community-dwelling women (60–81 years) who experienced BE in the past month	OBE diagnostic items from EDE-5	Of women reporting OBE ≥ 1/week for ≥ 3 months, 31.7% reported youth onset (age < 40), 17.1% reported midlife onset, and 51.2% reported late-life onset (age 56 +)	OBE prevalence = 77.5% OBE ≥ 1/week for ≥ 3 months prevalence = 57.7%	▪ Earlier binge eating onset ▪ Higher binge episode frequency ▪ Greater binge-related emotional distress
Jenkins PE et al., 2018 [65]	Cross-sectional study	Evaluating potential similarities and differences between women with EDs in midlife and their younger counterparts	703 women referred to an outpatient EDs service	DSM-V	Women aged 18–25 were less likely to be diagnosed with BED than other groups. Women aged 40 years and above were more likely to be diagnosed with BED Statistical analysis revealed significant differences among age groups regarding weight and shape concerns	BED prevalence (women aged ≥ 40): 13.2%	▪ Midlife women more frequently diagnosed with BED than younger ▪ Illness duration longer, onset later, symptom severity comparable across ages
Elran-Barak R et al., 2015 [58]	Cross-sectional study	i) Assessing similarities and differences between midlife and younger individuals with EDs; ii) Assessing similarities and differences among midlife individuals with EDs	2,118 eating disorder treatment-seeking adults aged 18 to 75 years at four outpatient eating disorder treatment centres; 364 participants were aged 40 years and above, of which 332 were female	EDQ version 8.1	Percentage of patients with BED was significantly higher among the midlife group than among the youngest group Midlife individuals with BED reported more frequently being married and having children than the younger counterpart Reported medical symptoms were higher among midlife individuals with BED rather than among younger participants	BED prevalence in women aged 40 + = 75/332 = 22%	▪ Midlife or older age ▪ Higher body mass index ▪ More compulsive exercising behaviours ▪ Different marital status patterns
Micali N et al., 2017 [59]	Two-phase study design (cohort study + cross-sectional study)	i) Assessing the lifetime and 12-month prevalence of EDs, investigating lifetime health service use; ii) identifying childhood, parenting, and personality risk factors	5542 women, average age = 47.78 years (SD: 4.5)	EDs section of the SCID-I (with no skip rules), supplemented with a version of the LIFE interview, adapted to EDs	Parental separation or divorce in childhood, child sexual abuse, childhood unhappiness, low maternal care reported, and parental overprotection were associated with increased odds for BED Amongst personality characteristics, a more external locus of control and higher interpersonal sensitivity were associated with increased odds for BED	BED weighted 12-month prevalence = 1.03% (0.73–1.46) Subthreshold BED weighted 12-month prevalence = 0.38% (0.22–0.67)	▪ Childhood unhappiness, ▪ Sexual abuse, ▪ Parental separation, ▪ Low maternal warmth, and ▪ Oppressive parental relationships
Khalil J et al., 2022 [61]	Cross-sectional study	Investigating the association between the transition to menopause, body dissatisfaction, and abnormal eating habits	1001 Lebanese women, aged 40 and above	BES	After adjusting for age, monthly income, BMI, marital status, education level, and body dissatisfaction, perimenopause was associated with more binge eating than premenopause	*n.r*	▪ Perimenopausal phase compared to premenopause ▪ Higher body dissatisfaction scores
Baker JH et al., 2019 [66]	Longitudinal study	Examining the interactive effect of changes in estradiol and progesterone on two cores, transdiagnostic eating disorder symptoms: binge eating and body dissatisfaction in midlife women during perimenopause	8 women aged 42–52 in early perimenopause who reported a history of at least two losses of control eating episodes in the month prior to study enrolment	EPSI	Interactions of daily E2 and P4 were observed for binge eating. Increasing E2 was strongly associated with higher BE when P4 was high, but was not associated with BE when P4 was low	*n.r*	Elevated estradiol in the context of high progesterone

For brevity and consistency with the aim of this narrative review, only findings related to BED and BE in the female population are reported

EDs, eating disorders; BE, binge eating; BED, binge eating disorder; BES, Binge Eating Scale; BMI, body mass index; BSQ, Body Shape Questionnaire; CESD, Center for Epidemiologic Studies Depression Scale; CI, Confidence Interval; DSM-IV, Diagnostic and Statistical Manual of Mental Disorders-IV; DSM-IV-TR, Diagnostic and Statistical Manual of Mental Disorders-IV—Revised Text; DSM-V, Diagnostic and Statistical Manual of Mental Disorders-Fifth Edition; E2, Estradiol; EDDS-5, Eating Disorder Diagnostic Scale for DSM 5; EDE-5, Eating Disorders Examination; EDE-Q, Eating Disorder Examination Questionnaire; EDI, Eating Disorder Inventory; EPSI, Eating Pathology Symptom Inventory; LIFE, Longitudinal Interval Follow-up Evaluation; OBE, objective binge episodes; OR, odds ratio; P4, progesterone; SCID, Structured Clinical Interview for DSM-IV; SCID-I, Structured Clinical Interview for DSM-IV-TR disorders; SD, standard Deviation; VA-BES, VA-Binge Eating Screener; n.r., not reported

**Table 4:** Binge eating and gender transition

Author and year	Type of study	Aim	Population	BED diagnosis/screening	Results	Prevalence	Associated factors
Nagata JM et al., 2020 [79]	Longitudinal cohort study	Helping to develop community standards for the EDE-Q, to assess disordered eating attitudes and behaviour, for the first time among transgender men and women	484 (TM/TF: 312/172); **TM:** 18–67 (years); **TF:** 20–74 (years)	EDE-Q (Fairburn & Beglin, 2008)	**TM:** Correlation between BMI and the subscales Eating Concern (r = .21, p < .001), Weight Concern (r = .34, p < .001) and Shape Concern (r = .25, p < .001) and with global score (r = .25, p < .001) Correlation with global score: Restraint (r = .58 to .87), Concern about food (r = .61 to .85), Concern about weight (r = .72 to .89), Concern about fitness (r = .68 to .86), and Global Score (r = .46 to .83) **TF**: Correlation between BMI and the subscales Eating Concern (r = .19, p = .014), Weight Concern (r = .39, p < .001), Shape Concern (r = .26, p = .001) and Global score (r = .28, p < .001) Participants with a higher BMI generally had higher EDE-Q scores: Restraint (r = .53 to .84), Food Concern (r = .54 to .83), Weight Concern (r = .71 to .89), Shape Concern (r = .63 to .86), and Global score (r = .40 to .82)	**TM:** 1.6% **TF:** 1.7%	Transgender identity (men, women) associated with objective binge episodes prevalence
Kramer R et al., 2024 [83]	Retrospective study	Describing and comparing the frequency and severity of EDs symptoms in TGD seeking gender-affirming treatment; exploring potential associations between the use of gender-affirming hormones and ED symptoms	251 (TM/TF: 181/70); TM: 12–24 (years), TF: 12–24 (years)	EDE-Q (Fairburn & Beglin, 1994)	**TM:** BMI: 26.87 kg/m^2^ ± 7.88 (min. 16.1- max 58.5); Subjective binge episodes: 15.6%; Objective binge episodes:17.6% **TF**: BMI: 25.05 kg/m^2^ ± 6.49 (min. 16.1- max 58.5); Subjective binge episodes: 23%; Objective binge episodes: 23.6% 50% of TF using gender affirmation hormones (n = 7, 50.0%) reported objective episodes of binge eating vs TM (n = 4, 10.8%), Fisher's Exact, p = 0.005, OR = 0.12, 95% CI [0.30, 0.53]; The association is significant between TF, the use of gender-affirming hormones and objective binge eating episodes, 1) = 5.54, p = 0.03, OR = 4.83, 95% CI [1.23, 18.98] Respectively, 17.1% (n.6) and 50% (n.7) of TFs who did not use hormones and who used gender-affirming hormones reported objective binge eating episodes	5.2% 19.2% reported at least one objective binge episode, 17.7% endorsed at least one subjective binge episode	In **TF:** ▪ Youth ▪ Current use of gender-affirming hormones
Arikawa AY et al., 2021[84]	Cross-sectional study	Describing the proportion of food insecurity and eating disorder behaviours in a volunteer sample of LGBTQ + individuals, and describing other outcomes such as health behaviours, anxiety and depression, stratified by gender identity classification	239 (M/F: 29/93; TM: 59; Gender non-conforming 58); 18–35 (years)	▪ EAT-26 (Ocker 2007) ▪ EDE-Q (Fairburn & Beglin, 1994)	81.8% used sex steroid hormones 64.8% reported food insecurity; high levels of depressive symptoms were detected in 91.4%; in 37.8% and 16.2% moderate and high levels of anxiety EDE-Q not statistically significant when stratified by gender EAT-26: inappropriate eating behaviours in 20.3% of the sample, up to 23.4% in the TM group	TM: 23.4% (EAT-26)	▪ Higher levels of food insecurity ▪ TM gender identity within the LGBTQ + sample ▪ Gender-nonconforming identity within the LGBTQ + sample ▪ Elevated depressive and anxiety symptoms co-occurring with disordered eating, including BE
Bell K et al., 2019 [90]	Cross-sectional study (as part of a national study conducted by the Laboratory for Resilience in Psychological and Physical Health in Johnson City, USA)	Studying and comparing the percentage of symptoms and predisposition to ED, understanding the factors that can be involved and evaluating how these variables interact with each other, in cisgender gay men, cisgender lesbian women and TGNC adult population	320 (Gay man:98; Lesbian women:84; TGNC Adults: 138) Mean age (SD): Gay man 42.54(± 17.19); Lesbian women 38.64(± 17.65); TGNC Adults 33.60(± 16.80)	Eating Disorders Screen for Primary Care (ESP) (Cotton et al., 2003)	Lesbian women vs gay men were more likely to have a higher ESP score (OR, 2.09; 95% CI, 1.08–4.05 Significant difference in terms of weight-based self-esteem, with lesbian women more likely than gay men (OR, 2.52; 95% CI, 1.19–5.37) and TGNC adults (OR, 2.44; 95% CI, 1.17–5.09) to endorse this item Lesbian women (OR, 2.83; 95% CI, 1.30–6.33) and TGNC adults (OR, 2.32; 95% CI, 1.10–4.88) are more likely than gay men to have a self-reported current or past experience of an eating disorder TGNC adults who were more likely to be dissatisfied with their eating patterns than gay men (OR, 2.30; 95% CI, 1.27–4.19)	*n.r*	▪ Higher depressive symptoms ▪ Lower self-compassion ▪ Greater perceived stigma and thwarted belongingness
Watson RJ et al., 2017 [86]	Exploratory study	Examining how varying combinations of risk and protective factors contribute to the probability of transgender youth engaging in disordered eating behaviours and examining four possible forms of social support (family, friend, school, and general social support) that have been identified as protective factors for youth	923 transgender youth; 14 -25 years	BE—*One yes/no item asked both 14–18 and 19–25-year-old youth: “During the past 12 months, have you eaten so much food in a short period of time that you felt out of control (binge eating)?”*	26% of the sample reported one disordered eating behaviour Experiences of stigma were positively associated with binge eating (p < 0.01); while the family and social connection scale were negatively (p < 0.01) [Odds ratio (95% CI)]	42% reported BE (at least once in the past 12 months)	▪ Higher enacted stigma (harassment, discrimination) ▪ Greater exposure to violence ▪ Lower family and school connectedness ▪ Fewer caring friends, weaker social support
Linsenmeyer WR et al., 2021 [88]	Cross-sectional study	Describing the prevalence and relationships among disordered eating, food insecurity, and weight status in transgenders and gender nonbinary youth and young adults	164 (TM:128/TF:28/ Nonbinary:8); 12–23 (years)	▪ SCOFF ▪ ADO-BED ▪ NIAS	8.7% reported a previous diagnosis of an ED; 28.0% tested positive on the SCOFF; 75.0% tested positive on the NIAS; 21.2% tested positive on the Hunger Life Sign Statistically significant positive correlation between BMI and ADO-BED (r = 0.25) Significant difference between gender identity groups on the NIAS (F = 4.58, p = 0.01): TM scored higher vs TF (p = 0.03) Significant negative correlation between BMI and NIAS scores in young people who transitioned with hormone therapy Significant effect of previous eating disorder diagnosis and Vital Hunger Sign scores (F = 3.08, p < 0.05)	9.1%	▪ Higher BMI ▪ Food insecurity
Pham AH et al., 2023 [89]	Longitudinal study	▪ Describe prevalence and frequency of disordered eating in TGNB adolescents over 12 months ▪ Examine trends in disordered eating by gender identity ▪ Assess longitudinal changes after initiation of gender-affirming medical care	91 (sex at birth M/F:31/69; gender identity TM:56; TF:29; nonbinary/fluid:6) Mean (± standard deviation) age = 15.2 ± 2.1 years	EDE-Q	62% were regularly dissatisfied with their shape 76% feel uncomfortable about their bodies Number of disordered eating behaviours reported by participants: at least 1 for 40% of participants (with a regular frequency for 22%) and at least 3 for 70% (with a regular frequency for 7%) No statistically significant differences for EDE-Q and gender identity, gender-affirming medication or time spent receiving gender-affirming treatment	26% of participants had BE episodes	Dissatisfaction with one’s shape/weight

kg, kilogrammes; m, metres; BMI, body mass index; EDs, eating disorders; TM, transgender male; TF, transgender female; EDE-Q, Eating Disorder Examination-Questionnaire; EAT-26, Eating Attitudes Test; ESP, Eating Disorders Screen for Primary Care; TGNC, Transgender and non-conforming adults; SCOFF, Sick, Control, One Stone, Fat, Food Questionnaire; ADO-BED Adolescent Binge Eating Disorder Questionnaire; NIAS, Nine-Item Avoidant/Restrictive Food Intake Disorder Screen; TGD, cisgender peers, transgender and gender diverse; TGNB, transgender and nonbinary

Yamamiya and colleagues [35] identified key predictors of future BED, including pressure to be thin, thin-ideal internalisation, negative emotionality, low parental support, and modelling of disordered eating behaviours, in a longitudinal cohort of adolescent girls followed for 8 years. Recurrent binge eating episodes may function as a coping mechanism to alleviate negative emotions associated with perceived low parental support in girls who later develop BED [35].

The cultural values embedded in media-portrayed beauty ideals contribute to body dissatisfaction and disordered eating behaviours in adolescents, highlighting the importance of interventions focused on media literacy, reducing self-objectification, and promoting emotional coping strategies [36]. Dakanalis and colleagues [37], in a longitudinal study of 685 adolescents, showed that internalisation of media ideals increases self-objectification, which in turn intensifies negative body-related emotions and promotes dietary restraint and binge eating, with consistent indirect effects across genders [37].

As reported by Bowlby J. [38], the attachment theory postulates that a child develops an early attachment bond with a specific caregiver, internalised as an internal representation of the self, other, and their mutual relationship. High levels of alexithymia are also common, characterised by difficulties in identifying and describing emotions [38]. Although both factors are considered transdiagnostic risk factors for BED, evidence in community populations remains limited [38].

In this context, Pace and colleagues [39], in a case–control study of adolescents girls, both preoccupied and dismissing attachment styles were independently associated with the severity of binge eating, suggesting a central role of attachment insecurity compared to alexithymia [39]. In a case–control study [40], girls with BE exhibited also lower narrative coherence (understood as the caregiver’s inadequate response to the child’s requests), weaker perceptions of their mother as a secure base, and greater anger towards their mother compared to controls [40].

Lastly, **childhood eating behaviours** appear to play a significant role in the development of eating disorders (EDs) and related behaviours during adolescence. To test the following hypothesis, Herle and colleagues [41] found that childhood overeating is associated with an increased risk of BE and BED during adolescence. Moreover, an increase in overeating during mid-to-late childhood predicts a higher likelihood of developing BED, suggesting that such behaviours may emerge at a very early age [41].

Summarising, BED risk factors during childhood and adolescence emerge from a complex interplay of psychological, sociocultural, and familial influences. Low self-esteem and body disinvestment are pivotal mechanisms, promoting self-objectification and negative emotional experiences these can drive dietary restraint and BE, irrespective of gender.

A lack of parental support, insecure attachment and limited access to food during childhood increase the risk, exacerbate vulnerability, hinder emotional regulation and encourage ‘emotional eating’.

With a view to preventing the onset of BED, programmes focusing on media literacy, the promotion of secure emotional bonds and the improvement of emotional management strategies that foster body acceptance and a positive body image should aim to strengthen resilience against cultural pressures and address dysfunctional family dynamics.

### Binge eating disorder and pregnancy

Pregnancy is a crucial stage of life, characterised by significant psychological, physiological, and behavioural changes that may contribute to the development of eating disorders, including BED, which is among the most frequently observed during this period [21, 42].

Prevalence estimates vary across studies: a prospective cohort study reported a prevalence of 4.8% at 17 weeks of gestation [48], while a systematic review conducted later, indicated lower rates (1.8%) [21].

A subsequent large-scale meta-analysis showed that BED is the most common ED before and during pregnancy (3.3% and 3.8%, respectively), with a significant increase during pregnancy compared to the pre-pregnancy period [43]. Additional data report lifetime and current prevalence rates of 1.67% and 0.51%, respectively [44].

BED during pregnancy is clinically relevant due to its impact on maternal and foetal health [44, 45]. It is associated with an increased risk of miscarriage and excessive gestational weight gain (GWG), which may lead to complications such as gestational diabetes, hypertensive disorders, caesarean delivery, postpartum weight retention, as well as adverse neonatal outcomes including large-for-gestational-age birth, childhood obesity, and metabolic disorders [45–47].

The factors associated with BED during pregnancy are still insufficiently studied; however, available evidence indicates a combined role of physiological adaptations (increased appetite and cravings), psychological and emotional factors, and social and lifestyle determinants [43, 48].

For instance, Knoph Berg analysed factors associated with the incidence and course of BED during pregnancy in 45,644 women from the Norwegian Mother and Child Cohort Study (MoBa) at approximately 18 weeks of gestation [48].

The incidence of BED was significantly associated with a history of sexual or physical abuse, major depression, symptoms of anxiety and depression, low life satisfaction, reduced self-esteem, poor partner relationship satisfaction, smoking, alcohol use, lack of social support, and various weight-related factors [48].

Persistence of BED during pregnancy was inversely associated with pre-pregnancy perceptions of being overweight [48]. Conversely, remission was positively associated with such pre-pregnancy perceptions and negatively associated with overvaluation of weight [48].

Finally, the multicentre cohort study LINDA-Brazil [47] examined BE during pregnancy in women with gestational diabetes and its association with GWG and postpartum weight retention. The prevalence of BE was high both during pregnancy (31.6%) and postpartum (30.0%). Women with BE had a 45% higher risk of exceeding GWG recommendations and a 33% higher risk of elevated postpartum weight retention compared to those without BE [47].

The management and identification of binge eating disorder (BED) during pregnancy can be hindered by a number of factors. These include women’s belief that they can exercise self-control without the need for professional help, a lack of awareness of what they are experiencing, and the failure to recognise BED as an eating disorder. Furthermore, stigma and the fear of being judged prevent pregnant women from speaking openly about their behaviours [44, 49].

At the same time, healthcare professionals do not always communicate effectively [49] or may lack education and training on how to identify and distinguish eating disorders [48, 49].

### Binge eating disorder in menopausal and postmenopausal women

Middle age is usually defined as the period between the ages of 40 and 60 [50]. According to several authors [51–53], the transition to the menopause, as a unique stage in a woman’s life, combined with concerns about ageing, body dissatisfaction and a distorted body image, may represent a period of increased vulnerability to psychological distress due to the complex physical, mental and social changes that occur and affect quality of life [22, 54, 55]. This situation may contribute to an increased risk of developing various chronic diseases, negatively affect mental health and also contribute to the development of EDs potentially influenced by transient hormonal fluctuations [55–57].

EDs have always been regarded as a problem affecting young people, but the evidence, although scarce and inconsistent, suggests that they are widespread among middle-aged and older women [51, 57–60]. Furthermore, there is a lack of awareness among healthcare professionals regarding the clinical manifestations of EDs in these specific populations, and the healthcare system falls short in supporting middle-aged and older women [61]. However, in recent decades, the rate of hospital admissions among middle-aged women seeking treatment for EDs has shown an upward trend [70]. Evidence suggests that EDs characterised by BE (such as BED) are more common among middle-aged and older adults than disorders characterised by food restriction and underweight [55, 58, 60, 62]. In this section, several studies [60, 61, 63–68] have examined the prevalence of BED and its associated risk factors among middle-aged and older women.

Overall, the studies indicate that BED is also present in middle and older age, with varying prevalence rates and frequent associations with psychological, social and body-related factors. Women with BED or BE often exhibit educational disparities and cultural differences, greater body dissatisfaction, depressive symptoms, internalised stigma and poorer general health, including a higher BMI and an increased risk of obesity [38, 65]. The onset of BE is often linked to stressful life events such as divorce, retirement or bereavement [66]. Clinical and population-based studies report a significant prevalence of BED among women over 40, with specific socio-demographic and clinical characteristics, including greater family responsibilities and less restrictive behaviours compared to younger age groups [60, 67].

In conclusion, eating disorders affecting middle-aged women and older are often unrecognised, untreated and chronic [69]. According to the population-based study conducted by Micali and colleagues [61], only 27.4% of participants with eating disorders had sought or received clinical care and treatment for an eating disorder at any point in their lives. In such cases, symptoms related to eating disorders may have become ego-syntonic due to the long duration of the disorder and the rigidity of habits. Due to their family and social roles, women may have little time to look after themselves, feeling shame and embarrassment about seeking healthcare [69]. Overall, BED is the most common eating disorder in middle age and during the menopause, driven by a complex interplay of sociocultural, psychological, hormonal and life-stage factors [53, 55, 64, 67, 70]. Routine screening for BED (in particular, the Screen for Disordered Eating–SDE) may be essential for timely diagnosis and intervention.

This review underlined a paucity of evidence regarding BED in midlife and beyond; notably, there is an urgent need for research addressing the best outreach and treatment programmes for adults [69].

### Binge eating disorder and gender transition

LGBTQ+ individuals refer to those who identify as Lesbian, Gay, Bisexual, Transgender, Queer or Questioning, along with other sexual and gender minorities [71, 72]. The " + " represents additional identities, such as asexual, intersex, pansexual, and more, reflecting the broad diversity within sexual orientation and gender identity [73]. These individuals may encounter unique health challenges, including elevated risks of mental health disorders, substance abuse, suicidal ideation, and barriers to healthcare access, often due to discrimination, stigma, and insufficient cultural competence among healthcare providers [74, 75]. To date, the health of LGBTQ+ populations constitutes a critical public health issue, primarily due to these disparities [74, 76]. Indeed, research consistently demonstrates that LGBTQ + individuals are at greater risk for specific physical and mental health concerns [74], including HIV/AIDS [77, 78]; depression, anxiety, violence-related trauma [79], and EDs, including BED [25]. Furthermore, transgender individuals face significant challenges in accessing gender-affirming healthcare, which mainstream medical systems frequently fail to address adequately [80]. Therefore, public health initiatives must consider the social, cultural, and institutional factors that contribute to these disparities to ensure equitable healthcare access and improve health outcomes for LGBTQ + individuals.

Studies focusing on the transgender population, particularly concerning adverse health outcomes and the development of EDs, remain limited and fragmented [81]. Nevertheless, the association between transgender identity and heightened risk of EDs is well-established and largely stems from the pervasive stigma and discrimination faced by these individuals [24, 25]. Experiences of stigma contribute to minority stress, exacerbated by societal pressures to conceal gender identity or conform to gender norms, such as the thin ideal for women or the muscular ideal for men [24, 25]. Specifically, In particular, Meyer proposed the ‘Minority Stress Model’, according to which sexual minorities are exposed to stress as a result of stigmatisation [82]. This is thought to contribute to the development of psychopathology [83]. These pressures often trigger disordered eating behaviours aimed at achieving alignment with the desired gender presentation or suppressing secondary sex characteristics associated with the sex assigned at birth [24, 25]. As a result, transgender individuals are at a significantly higher risk of developing EDs compared to their cisgender peers [25, 84]. This elevated risk is associated with severe physical and mental health consequences, increased healthcare costs, and heightened mortality rates from various causes, including suicide [24, 85, 86].

The paragraph summarises findings from seven studies on risk and protective factors for BED in transgender individuals, highlighting considerable variability in the prevalence of EDs and BE. Among transgender and nonbinary adolescents, prevalence ranges from 5 to 18%, while in transgender adults EDs more broadly may affect 20–50% [87–89]. Reported BED prevalence varies widely across studies and populations, from 1.6–1.7% in the PRIDE cohort studies [81] to 9.1% in transgender youth [88], and up to 42% of BE episodes in a large Canadian survey of transgender adolescents [86], reflecting substantial methodological heterogeneity.

Key risk factors include body dissatisfaction, higher BMI, and concerns about weight and shape, which are consistently associated with BED across studies [81, 90, 91]. Food insecurity is also a significant contributor and is linked to depressive and anxiety symptoms as well as BE behaviours [86]. Gender-affirming hormone therapy shows complex associations with BE episodes, with differences observed between transgender men and women [85].

Among social factors, stigma emerges as a central determinant of vulnerability, while self-compassion and supportive family and school environments act as important protective factors [88, 92].

In conclusion, BED in transgender individuals is shaped by a combination of psychological, body-related, social, and structural factors. Its management requires an integrated approach that includes gender-affirming care, psychological interventions, anti-stigma strategies, and systemic measures to address food insecurity, alongside strengthening protective social environments [75, 81, 86, 88, 93–95].

Minority stress, body dissatisfaction, stigma, discrimination and pressure to conform to gender norms may interact synergistically in increasing BED vulnerability among transgender individuals.

## Conclusions

BED is an important concern for the public and mental health [3]. Our review highlights how BED in the female population is strongly influenced by both, sex-related biological changes and gender-based sociocultural factors. Simultaneously, sociocultural pressures—such as body dissatisfaction, low self-esteem, and internalised weight stigma—further exacerbate the risk of BED onset and persistence [35, 64]. These determinants often stem from media-driven beauty ideals and societal constructs of femininity [33, 35–37, 48, 64, 65], making BED not only a medical issue but also a reflection of broader cultural influences. The prevalence of binge eating appeared to increase across the lifespan, with rates averaging 11% among adolescents aged 13–18 years, 20% among pregnant women, and 30% among women undergoing menopause. In transgender populations, the reviewed studies reported the highest prevalence during adolescence and young adulthood, possibly reflecting the combined influence of identity affirmation processes, the pursuit of gender-related change, and exposure to minority stress.

To ensure health equity, clinical research must be inclusive and consider the specific vulnerabilities of marginalised groups [18]. In this context, transgender individuals. unique and significant barriers to BED diagnosis and treatment [74]. Transgender women and men experience structural stigma at societal, cultural, and political levels, resulting in poorer health outcomes [86]. This population shows a heightened risk of BED, driven by minority stress, food insecurity, gender-affirming hormone use, and body image concerns [81, 90, 91].

Despite increasing awareness, our review reveals significant research gaps, particularly regarding certain high-risk populations. The available studies remain fragmented and lack standardised methodologies, limiting the generalisability of findings. There is an urgent need for more comprehensive research to develop tailored, holistic interventions and improve healthcare providers’ preparedness. Overcoming the stigma and shame experienced by individuals with BED, especially women and transgender individuals, requires systemic change at both the clinical and policy levels [24, 25, 44, 49, 69, 75].

Binge eating disorder (BED) should be prioritised in the global and public health agenda, given its strong links to chronic diseases such as obesity [65] and depression [48]. In pregnant women, BED represents a key risk factor for excessive gestational weight gain and postpartum weight retention [45, 47]. Policymakers play a crucial role in shaping environments that reduce BED risk, emphasising prevention, awareness, and equitable access to treatment.

Addressing BED effectively requires multidisciplinary collaboration, early intervention, and the dismantling of societal stigmas surrounding disordered eating and body image.

Finally, the included studies showed substantial heterogeneity in study design, sample characteristics, age ranges, cultural and geographical settings, and diagnostic approaches, limiting direct comparability across findings. BED was assessed using different diagnostic criteria (DSM-IV, DSM-5, subthreshold definitions) and a wide range of screening and self-report instruments, potentially contributing to variability in prevalence estimates and associated risk factors. Additionally, many studies relied on cross-sectional methodologies, self-reported measures, or relatively small and non-representative samples, reducing the possibility of establishing causal relationships and limiting the generalisability of results. Differences in the operationalisation of psychological and sociocultural constructs, such as body dissatisfaction, stigma, emotional dysregulation, and attachment insecurity, further complicate the integration of findings across developmental stages. Another limitation concerns the uneven distribution of evidence among the populations examined, with some groups, particularly transgender individuals and women in midlife or late adulthood, remaining underrepresented in the literature. Finally, as this is a narrative review, the methodological approach does not allow for quantitative synthesis or formal assessment of study quality and risk of bias, which should be considered when interpreting the conclusions of the review.

## Data Availability

No datasets were generated or analysed during the current study.

## Abbreviations

AIDS: Acquired immune deficiency syndrome
APA: American Psychiatric Association
BE: Binge eating
BED: Binge eating disorder
BES: Binge Eating Scale
BMI: Body mass index
CBT: Cognitive behavioural therapy
CBT-E: Enhanced cognitive behavioural therapy
DSM-IV/5: Diagnostic and Statistical manual of mental disorders-IV/5
EDs: Eating disorders
EDI: Eating Disorder Inventory
FFI: Friends and family interview
GDM: Gestational diabetes mellitus
GWG: Gestational weight gain
HIV: Human immunodeficiency virus
IOM: Institute of Medicine's
IPT: Interpersonal psychotherapy
LGBTQ: Lesbian, gay, bisexual, transgender and queer
NCDs: Non-communicable diseases
OBE: Objective binge eating
PEBS: Prenatal Eating Behaviours Screening
SDE: Screen for Disordered Eating
SKID: German version of the Structured Clinical Interview for DSM-IV
TAS-20: Toronto Alexithymia Scale
